# On-Demand Hospital Manufacturing of Baclofen Lozenges Using Pharmaceutical 3D Printing

**DOI:** 10.1007/s11095-026-04164-7

**Published:** 2026-08-11

**Authors:** Daniel Grandal-Gonzalez, Carlos Bendicho-Lavilla, Eduardo Diaz-Torres, Rocio Sanin-Fontela, María L. Couce, María José de Castro-López, Isabel Gonzalez-Alvarez, Marta Gonzalez-Alvarez, Alejandro Ruiz-Picazo, Marival Bermejo, Pedro Garcia-Salom, Abdul W. Basit, Carmen Alvarez-Lorenzo, Alvaro Goyanes

**Affiliations:** 1 Departamento de Farmacología, Farmacia y Tecnología Farmacéutica, I+D Farma (GI-1645), Facultad de Farmacia, Materials Institute (iMATUS) and Health Research Institute of Santiago de Compostela (IDIS), Universidade de Santiago de Compostela, 15782 Santiago de Compostela, Spain; 2 FABRX Artificial Intelligence, Enrique Vidal Abascal 7, 15702 Santiago de Compostela, Spain; 3 FABRX Ltd., Henwood House, Henwood, Ashford, Kent, TN24 8DH UK; 4 Servicio de Neonatología, Unidad de Diagnóstico y Tratamiento de Enfermedades Metabólicas Congénitas, Health Research Institute of Santiago de Compostela (IDIS), Hospital Clínico Universitario de Santiago de Compostela, Universidad de Santiago de Compostela, IDIS, RICORS, CIBERER, MetabERN, Santiago de Compostela, Spain; 5 Department of Pharmacokinetics and Pharmaceutical Technology, Miguel Hernandez University, 03550 San Juan de Alicante, Spain; 6 Pharmacy Service, Dr. Balmis General University Hospital, 03010 Alicante, Spain; 7 Department of Pharmaceutics, UCL School of Pharmacy, University College London, 29-39 , Brunswick Square, London, WC1N 1AX UK

**Keywords:** impaired swallowing function, personalized pharmaceuticals, pharma-ink, process analytical technologies, semi-solid extrusion (SSE) 3D printing

## Abstract

**Purpose:**

Dysphagia in neurological patients complicates oral drug administration. Manual preparation of lozenges/troches lacks precision and scalability. This study investigates the feasibility of implementing a pharmaceutical 3D printer in a hospital pharmacy for the automated production of personalized baclofen troches.

**Methods:**

Baclofen troches were manufactured in a hospital pharmacy setting, both manually and via pharmaceutical 3D printer. Quality was evaluated using Process Analytical Technologies (PAT). Performance was assessed through European Pharmacopoeia (EP) tests for drug content, mass uniformity, *in* *vitro* dissolution, disintegration, and a six-month stability study.

**Results:**

Printed troches complied with all EP acceptance criteria for drug content and mass uniformity, whereas manual formulations failed, particularly at the lowest dose. Printed troches demonstrated immediate drug release, consistent quality across batches, and remained stable for six months. 3D printing showed superior precision over traditional manual compounding.

**Conclusions:**

This research describes the first successful implementation of an automated blister-filling workflow using a pharmaceutical 3D printer within a hospital pharmacy setting, spanning from manufacturing and dispensing to direct patient administration. The automated approach provides a significant advancement in personalized medicine, ensuring safer, more accurate, and efficient treatment options for patients with specific needs like dysphagia.

**Supplementary Information:**

The online version contains supplementary material available at https://doi.org/10.1007/s11095-026-04164-7.

## Introduction

Dysphagia is defined as a symptom of dysfunction that occurs between the mouth and stomach, characterized by difficulty swallowing both solid and liquid foods [1]. The prevalence of this condition is 11% in the general population, rising to 25% in people over 70 years of age [2]. There are several types of dysphagia depending on the possible causes, evaluation or treatment [3]. One of the most common causes of this condition is the presence of a muscular or neurological disorder, which directly or indirectly affects the ability to swallow. Therefore, it is quite common for patients with conditions such as Parkinson’s, multiple sclerosis, or amyotrophic lateral sclerosis to also present with dysphagia [4].

Currently, most of the drugs used to produce systemic effects are administered orally. This route is the most popular due to numerous factors, such as the ease of administration of the dosage form, its high versatility, and its low production cost [3]. However, for patients with dysphagia, certain dosage forms, such as tablets or capsules, are difficult to swallow [5]. The coexistence of impaired swallowing function and inadequate dosage design contributes to a poor experience for this group of patients, negatively impacting treatment adherence [6]. Consequently, it is common for patients to manage these difficulties by altering the therapeutic regimen. Many of them skip doses or modify the administration method by crushing tablets or opening capsules, which can lead to significant dosing errors [7, 8].

Compounding is defined as the practice in which a professional combines, mixes, or alters the composition of a drug to suit a patient's individual needs [9]. The process emerges to meet patient requirements that cannot be fulfilled by the medicines provided by the pharmaceutical industry [10]. In recent years, there has been a growing demand for compounded medicines, due to dose limitations, the combination of active principle ingredients (APIs), or the need for alternative organoleptic characteristics [11]. However, this practice carries several risks. Unlike commercially manufactured medicines, compounded preparations are not subject to the same stringent regulatory oversight. Neither the United States Food and Drug Administration (FDA) nor the European Medicines Agency (EMA) is directly involved in the approval of compounded products. Consequently, these formulations are not required to meet the same regulatory standards for demonstrating safety and efficacy as industrially produced medicines [12]. Moreover, in many countries, compounded products are not mandated to comply with the guidelines set forth by Good Manufacturing Practices (GMP) [13]. As a result, numerous incidents have been reported, ranging from therapeutic ineffectiveness to patient fatalities [14, 15]. These concerns underscore the urgent need for substantial improvements in compounding practices and the implementation of rigorous quality control measures.

Troches or lozenges are a common dosage form prepared through compounding. These are solid formulations that contain one or more drugs, typically within a pleasant-tasting matrix with a sweetened base [16]. They are generally prepared through compression or molding, allowing for the production of personalized formulations through a relatively simple process [17]. Troches are considered an alternative to other dosage forms when medication customization is required, such as for patients with dysphagia. Their ability to dissolve or disintegrate slowly in the mouth makes them particularly suitable for patients with swallowing difficulties, enabling drug administration by the oral cavity [16]. However, the manual preparation of this dosage form presents several challenges. The molding process must be carried out while the formulation is in a liquid state, requiring the molds to be filled quickly to prevent premature solidification, which could affect the quality of the final dosage form. Additionally, manual compounding of troches limits hospital capacity to scale production, as the process, although seemingly straightforward, requires precise technique and meticulous attention to detail [18].

Three-dimensional printing (3DP) technology for pharmaceuticals has emerged as a promising approach to personalized medicine. It relies on the additive layer-by-layer deposition of materials to create three-dimensional structures with precise geometries [19]. This innovative technique offers numerous advantages for drug development, including the customization of dosage, drug combinations, organoleptic properties, and release profiles, among others [20, 21]. Numerous clinical studies confirm the viability and efficacy of 3D printing in pharmaceuticals, with satisfactory results across various patient populations [22–24]. Despite its promising potential, many healthcare professionals remain unfamiliar with the applications of 3D printing, including the various techniques available and the range of dosage forms that can be produced [20, 25–28].

User-friendly approaches such as automated filling, which are easy to implement in pharmaceutical practice, have the potential to bridge this gap and make 3D printing more accessible to pharmacists. In a recent study, a 3D printer was employed to produce minoxidil capsules in a community pharmacy, resulting in notable improvements in workflow efficiency, cost reduction, process safety, and dose uniformity of the capsule [29]. This highlights the feasibility of this technology at dispensing points for the on-demand production of personalized medications. A 3D pharmaceutical printer would enable a faster and automated production of troches using molds or blister filling, allowing the obtention of personalized doses with integrated quality control. The combination of 3D printing of medicines with process analytical technologies (PAT), such as an analytical balance or a pressure sensor could improve the quality of the medicines, facilitating the adoption of this technology.

The aim of this study is to evaluate the feasibility of implementing an automated blister-filling workflow using a pharmaceutical 3D printer within a hospital pharmacy setting for the production of personalized baclofen troches at varying doses. Baclofen is a GABA-B receptor agonist commonly used for the management of spasticity in patients with neurological disorders. Many of these patients experience dysphagia, making conventional tablet administration unsuitable for their needs [30]. This manufacturing approach seeks to improve the compounding process by enhancing speed, efficiency, and integrated quality control. Furthermore, manually prepared troches were compared to those produced using 3D printing to assess differences in dose accuracy and production time.

## Materials and Methods

### Materials

Baclofen was obtained from Fagron (Barcelona, Spain). Techna 20 SF™ Troche Base, Orange Citrus™ Natural SD Powder Flavor Concentrate, and Mandarin Orange Natural Food Color Powder were obtained from SpecializedRx (Circle Pines, MN, USA).

### Automated Preparation of Baclofen Troches Using a Pharmaceutical 3D Printer

#### Pharma-ink Preparation

The pharma-ink, a mixture of the API and excipients ready for printing, was prepared following a protocol designed to enable seamless implementation in hospital pharmacies. Standard Operating Procedures (SOP) were followed to weight the required amount of each component (w/w): 96.8% Techna 20 SF™, 1.7% baclofen, 1.0% flavoring, and 0.5% coloring. Briefly, the Techna 20 SF™ base was placed in a metallic container and heated to 50°C. Once melted, baclofen, along with the coloring and flavoring agents, was incorporated under magnetic agitation, and the stirring continued for 10 min. After achieving a homogeneous mixture, the magnetic stirrer was removed, and the resulting pharma-ink was transferred to a disposable 20 mL Injekt® Luer Solo syringe (B. Braun Medical AG, Germany). Finally, the syringe was left at room temperature until the mixture solidified.

#### Printing Process

**Fig. 1:**
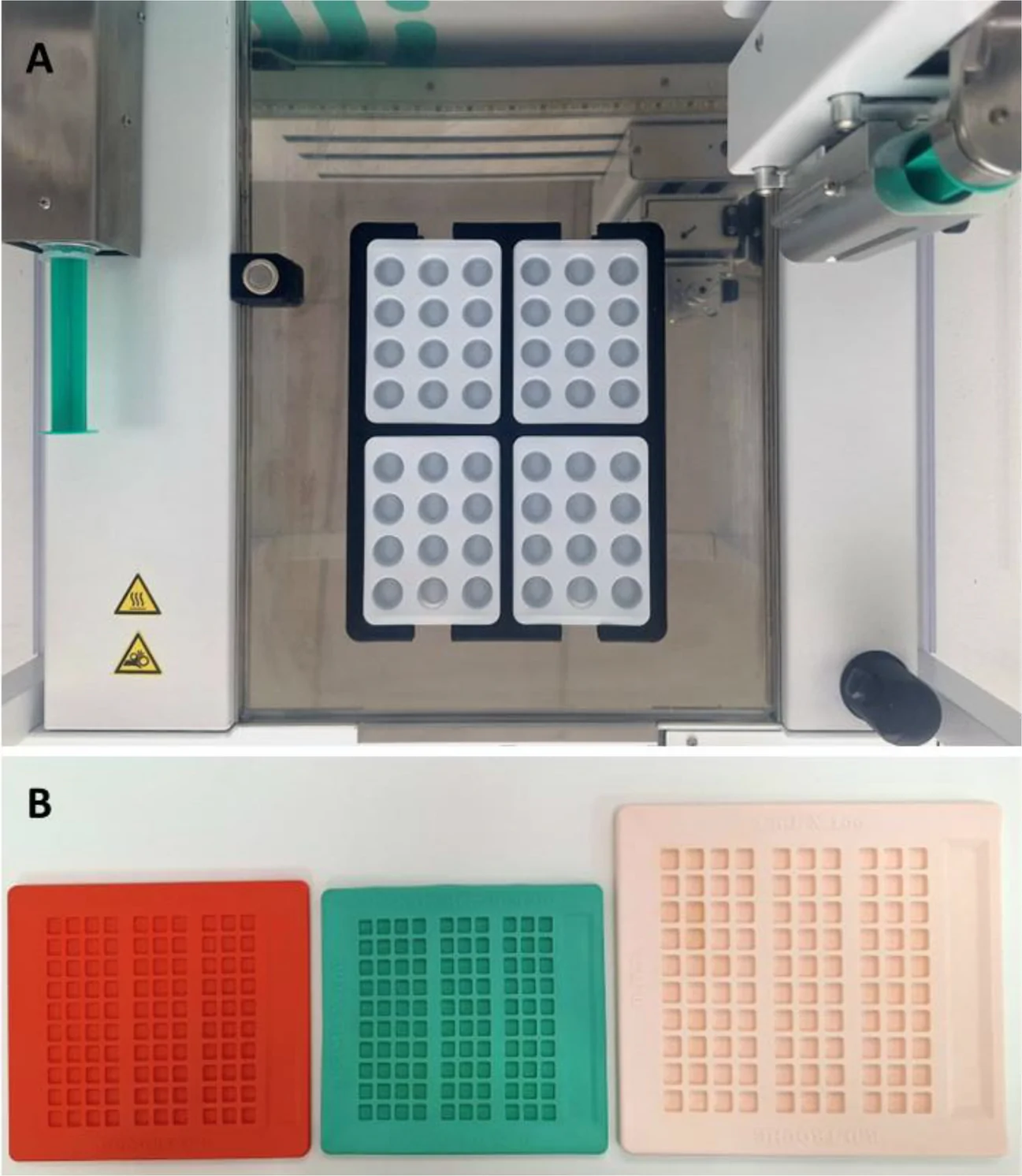
(**A**) Blister holder in the 3D printer, capable of holding up to four blisters at a time; (**B**) Silicone molds with different cavity volumes (0.25 mL, 0.5 mL and 1 mL, from left to right, respectively) used for the manual preparation of troches.

The troches were printed via a blister-filling process using a M3DIMAKER 1™ (FABRX, UK) pharmaceutical 3D printer with a semi-solid extrusion (SSE) Laguna printhead. Pre-loaded disposable syringes containing pharma-inks, fitted with a 1.2 mm diameter metallic nozzle (Fisnar, USA), were placed in the printhead. The printhead was heated to ensure the inks reached an appropriate viscosity for the extrusion process into the blisters. Size E blisters (Microcaya, Spain) were placed in a blister holder, specially designed for this technology (Fig. 1A).

Before initiating the printing process, both the pharma-ink and the blisters were registered in the M3DIMAKER Studio™ software (FABRX Ltd., UK) that controls the printer. The manufacturing process was initiated after selecting the appropriate blister, the dose of the troches and the number of troches per batch. The M3DIMAKER Studio™ software employs internal algorithms to correlate the weight of the pharma-ink extruded into the blisters with the desired dosage, ensuring the dose accuracy. Troches with doses of 5, 10, and 20 mg of baclofen were prepared. Finally, the loaded blisters were heat-sealed using the EASYPACK MINI blister sealing machine (Ataena, Italy).

#### In-line Quality Control

To ensure the quality of the troches, the 3D printer was equipped with multiple quality control systems. The integrated balance measured the weight of each troche in real time during the extrusion process. A color-coded system in the M3DIMAKER Studio™ software indicated whether the weight was within (green) or outside (red) the acceptable limits of a 5% deviation from the target, according to deviation requirements of the European Pharmacopeia (EP). Additionally, the SSE Laguna extrusion printhead included a pressure sensor that enabled the assessment of the rheological properties of the pharma-ink, facilitating the optimization of the printing parameters [31].

### Manual Preparation of the Troches

#### Preparation Process

The same proportion of excipients and API used for the automated preparation of troches was used for the manual preparation. Techna 20 SF™ was placed in a container and heated in a water bath at 45–50 °C. Once fully melted, the remaining components were added, and the mixture was stirred until a homogeneous, lump-free consistency was achieved. The mixture was manually poured into the molds (Tickerworks, USA) and evenly distributed across all cavities using a spatula. Three molds of different capacities were used: a 0.25 mL mold for the 5 mg dose, a 0.5 mL mold for the 10 mg dose, and a 1 mL mold for the 20 mg dose (Fig. 1B). Once dried, the troches were demolded and packed in single-dose blisters (Dextromedica SL, Spain) and sealed manually using an adhesive based cold-sealing method.

### Characterization

#### Pharma-ink Extrudability

The extrudability profile of the pharma-ink was studied to determine the optimal printing parameters. The pharma-ink was heated at various temperatures (45, 50, 55 and 60 °C) and evaluated at different extrusion speeds (50, 60 and 70 mm/min) to achieve an appropriate viscosity and ensure process optimization. Characterization was performed using the SSE Laguna printhead, equipped with an integrated pressure sensor, while experimental conditions were configured via M3DIMAKER Studio™ software. Three distinct stages were tested: an extrusion period (first 50 s) to determine the threshold pressure required for steady-state flow; a holding period (60 s) to monitor the material's response following the cessation of applied force; and a retraction period (5 s) to counteract the viscoelastic recovery of the pharma-ink. Additionally, the relation between the plunger displacement and the extruded weight was evaluated to determine the extrusion rate, which the software uses to create the dose library.

#### Mass Uniformity

The mass uniformity of both 3D printed and manually prepared troches was evaluated in accordance with the EP monograph [32]. Twenty troches of each dose were weighed, and the mean and standard deviation were calculated. For the printed troches, an additional automated mass uniformity test was performed using the printer´s integrated balance.

#### Dimensions of the Troches

The dimensions of the troches were evaluated to verify uniformity in shape and size. For this purpose, the height, width, and length of 20 troches were measured using a digital vernier calliper 150 mm (eSynic, China). Subsequently, the mean and percentage deviation were calculated for each dosage. The results are considered acceptable when the percentage deviation was below 5%.

#### Friability Test

The friability of the troches was determined in accordance with the standards established in the EP [33]. For this purpose, 10 troches of each dose were randomly selected, weighed, and placed in the drum of a PTF 10ER friabilator (Pharma Test, Germany). The analysis was carried out for 4 min at a speed of 25 rpm. Afterwards, the troches were reweighed, and the weight loss was calculated considering the initial weight (P1) and the final weight (P2), as indicated in the following Eq. (1)

1 $$\%\;weight\;loss=(P1-P2)/P1x100$$

The results are considered acceptable when the percentage of weight loss does not exceed 1%.

#### X-ray Powder Diffraction Analysis (XRPD)

The diffractograms of powder ingredients (baclofen, flavoring and colorant), pharma-ink and troches were obtained in a D8 Advanced diffractometer (Bruker, Billerica, MA, USA) equipped with a sealed X-ray tube (CuKα1; λ = 1.5406 Å) and a LYNXEYE-2 detector. The voltage and the intensity used during the analysis were 40 kV and 40 mA, respectively. The diffractograms were obtained in the 2θ angular range of 3–40° with a step of 0.02° and a counting time of 2 s per step. Samples were spun to minimize the effect of the preferential orientation and were deposited on an oriented Si (511) plate to avoid scattering noise.

#### Thermal Analysis

Differential scanning calorimetry (DSC) was used to analyze the thermal behavior of the excipients and drug, pharma-ink, and troches. Samples (1–6 mg) were heated in a DSC Q200 (TA Instruments, New Castle, DE, USA) at a heating rate of 10 °C/min until 300 °C, using nitrogen as the purge gas at a flow rate of 50 mL/min.

Thermogravimetric analysis (TGA) was carried out using a TGA55 Discovery Series (TA Instruments, New Castle, DE, USA) for samples (3–9 mg) heated from 25 to 350 °C at a rate of 10 °C/min. Nitrogen was used as the purge gas at a flow rate of 50 mL/min.

#### Determination of Drug Content

The baclofen content in the troches was determined using a high-performance liquid chromatography-ultraviolet (HPLC–UV) method. The analysis was performed using an Agilent 1100 Series system equipped with a Waters Spherisorb column (ODS2 3 µm, 4.6 × 100 mm), maintained at 40 °C. An injection volume of 20 µL was used with a flow rate of 1.2 mL/min. The mobile phase consisted of a mixture of methanol and 0.05% TFA in water in a 40:60 v/v ratio. Detection was carried out at a wavelength of 266 nm, and baclofen exhibited a retention time of 3 min. The method was validated and demonstrated to be adequate regarding linearity, accuracy, precision and repeatability. The limit of detection (LOD) of baclofen was 0.94 µg/mL and the limit of quantification (LOQ) was 2.85 µg/mL.

Three troches of each dose were randomly selected and dissolved in methanol:water (50:50 v/v). The solution was centrifuged, and the drug content was quantified using Eq. 2. The test was carried out for printed and manual troches.

2 $$Drug\; content\left(\%\right)=\frac{Measured\;amount\;of\;baclofen\; in\;troche\;(mg)}{Theoretical\;dose\;of\;baclofen\;in\;troche\;(mg)}\times100$$

#### *In vitro* Disintegration Test

*In vitro* disintegration time was evaluated for both types of troches using a disintegration apparatus (Turu Grau, Spain) consisting of a basket-rack assembly compliant with the EP [34]. The test was performed in 900 mL of phosphate buffer (pH 6.8 ± 0.5) maintained at 37 ± 0.5 °C. Samples were subjected to constant agitation at 30 cycles per minute, and plastic discs were added to prevent floating of the troches. The test was performed in triplicate [35].

#### *In vitro* Dissolution Test

The release of baclofen was determined under conditions simulating possible swallowing of the troche, according to the United States Pharmacopoeia and National Formulary (USP-NF) baclofen tablets monograph [36]. Accordingly, the tablets were placed in the vessels of a Plus Dissolution Test Station USP Type II apparatus (Hanson Research, USA). A 0.01 N HCl solution was used as the dissolution medium. A volume of 500 mL was used for the 5 mg and 10 mg doses, while 1000 mL was used for the 20 mg dose, as indicated in the USP-NF. The test was conducted at 50 rpm and a temperature of 37 °C. Samples of 5 mL were withdrawn every 5 min over the course of one hour and immediately replaced with fresh medium. Finally, the samples were filtered using 0.22 µm filters (Millipore Ltd., Ireland), and drug concentration was determined using the previously described HPLC method.

#### Stability Test

Six-month stability study was conducted on printed and manual troches, according to the ICH guidelines *‘Q1A(R2) Stability Testing of New Drug Substances And Products’ *[37]. The troches were stored in a climatic chamber at 25 °C ± 2 °C and 60% RH ± 5% RH. Samples were analysed at 0, 15, 30, 60, 90, 120, and 180 days using the HPLC method described in Sect. "Determination of Drug Content"*.*

### Preparation and Dispensing of Baclofen Troches Using a Pharmaceutical 3D Printer in a Pharmacy Hospital

The M3DIMAKER 1 pharmaceutical 3D printer was installed in the Pharmacy Department of the General University Hospital Doctor Balmis (Alicante, Spain). Comprehensive regulatory and technical documentation was provided by FABRX Ltd. and FABRX Artificial Intelligence (FABRX AI), including Factory Acceptance Testing (FAT), Installation, Operational and Performance Qualifications (IQ/OQ/PQ), and Site Acceptance Testing (SAT). Furthermore, SOPs were established for the 3D printer’s operation, software interface, and cleaning protocols, alongside newly developed SOPs for pharma-ink preparation and the blister-filling process.

The pharmacists responsible for compounding were trained in the preparation of the pharma-inks, the use and handling of the printer, and the operation of the associated software. Printed troches containing 5, 10, and 20 mg of baclofen were prepared and dispensed to patients according to their prescriptions over the period of three months.

## Results and Discussion

This study aimed to implement a pharmaceutical 3D printer in a hospital setting for the automated production of baclofen troches. Baclofen troches of varying doses were prepared using both the traditional compounding and automated 3D printing processes. Both types of troches were compared based on their physicochemical properties, drug release profiles, and long-term stability.

The formulation components were selected based on their extensive use in pharmaceutical compounding and well-established safety profiles. The primary excipient of the troches was the commercial base Techna 20 SF™, a blend of different grades of polyethylene glycols (PEG) which acts as a polymeric matrix and provides structural integrity; stevia as a sweetener; xanthan gum as a suspending agent; and a bitter taste-masking agent. All components are classified as Generally Recognized as Safe (GRAS). Furthermore, the solid state of this base at room temperature, combined with its relatively low melting point, makes it particularly suitable for 3D printing technologies [38].

**Fig. 2:**
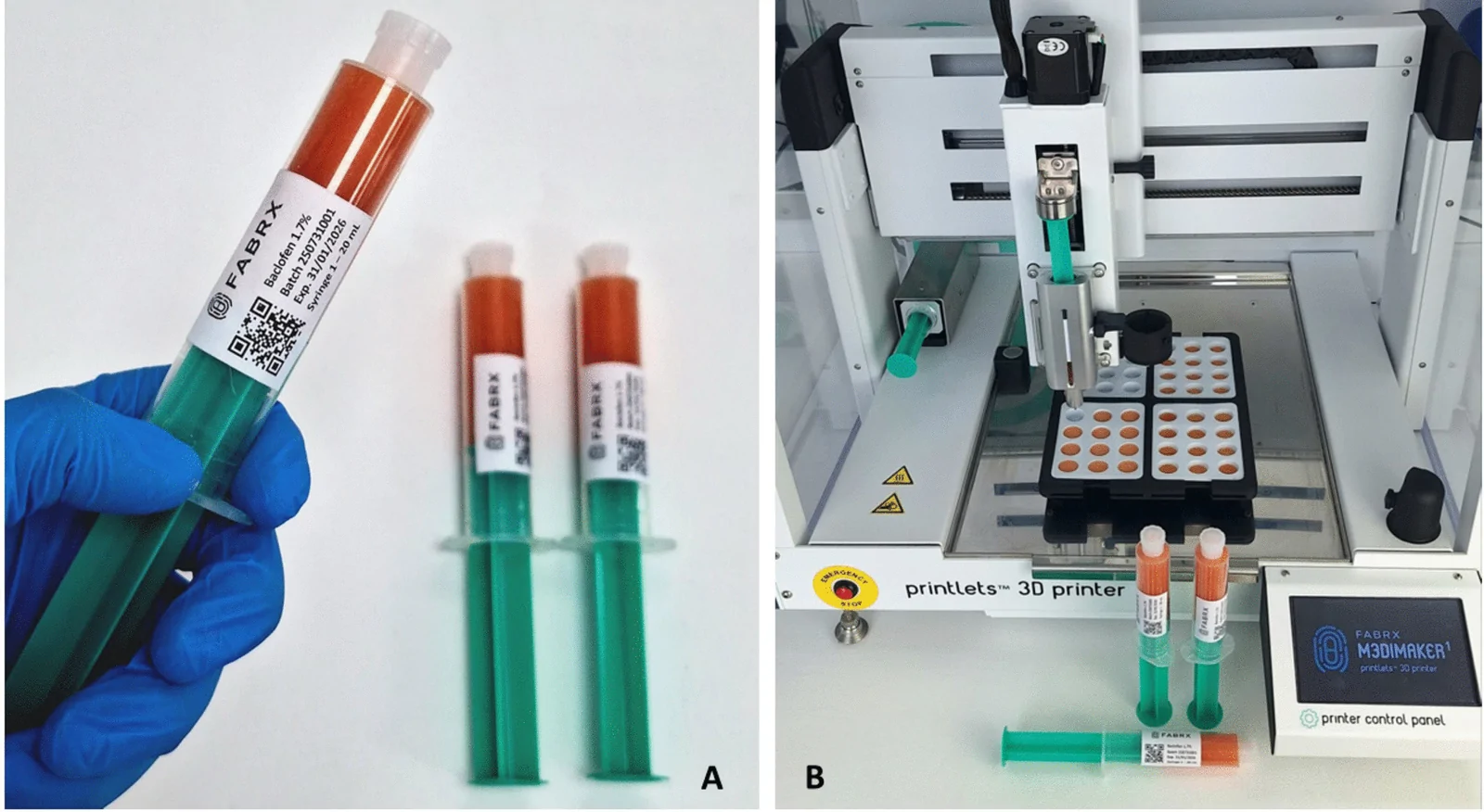
(**A**) Syringes filled with the pharma-ink, labeled with QR codes; (**B**) Automated blister-filling process using the M3DIMAKER 1 pharmaceutical 3D printer equipped with balance and pressure sensor.

The use of preformulated bases in compounding enhances pharmacists' workflow efficiency, as they only need to melt the base and incorporate the API, along with a colorant and/or flavoring agent if required. Both the colorant and flavoring agents used in the present study were composed entirely of natural ingredients, offering a favorable alternative to synthetic compounds [39, 40]. The preparation of the pharma-ink was straightforward, rapid, and reproducible. The resulting formulation was homogeneous, with no visible aggregation or clumps. The syringes containing the pharma-ink were labeled with QR codes to facilitate traceability (Fig. 2A).

**Fig. 3:**
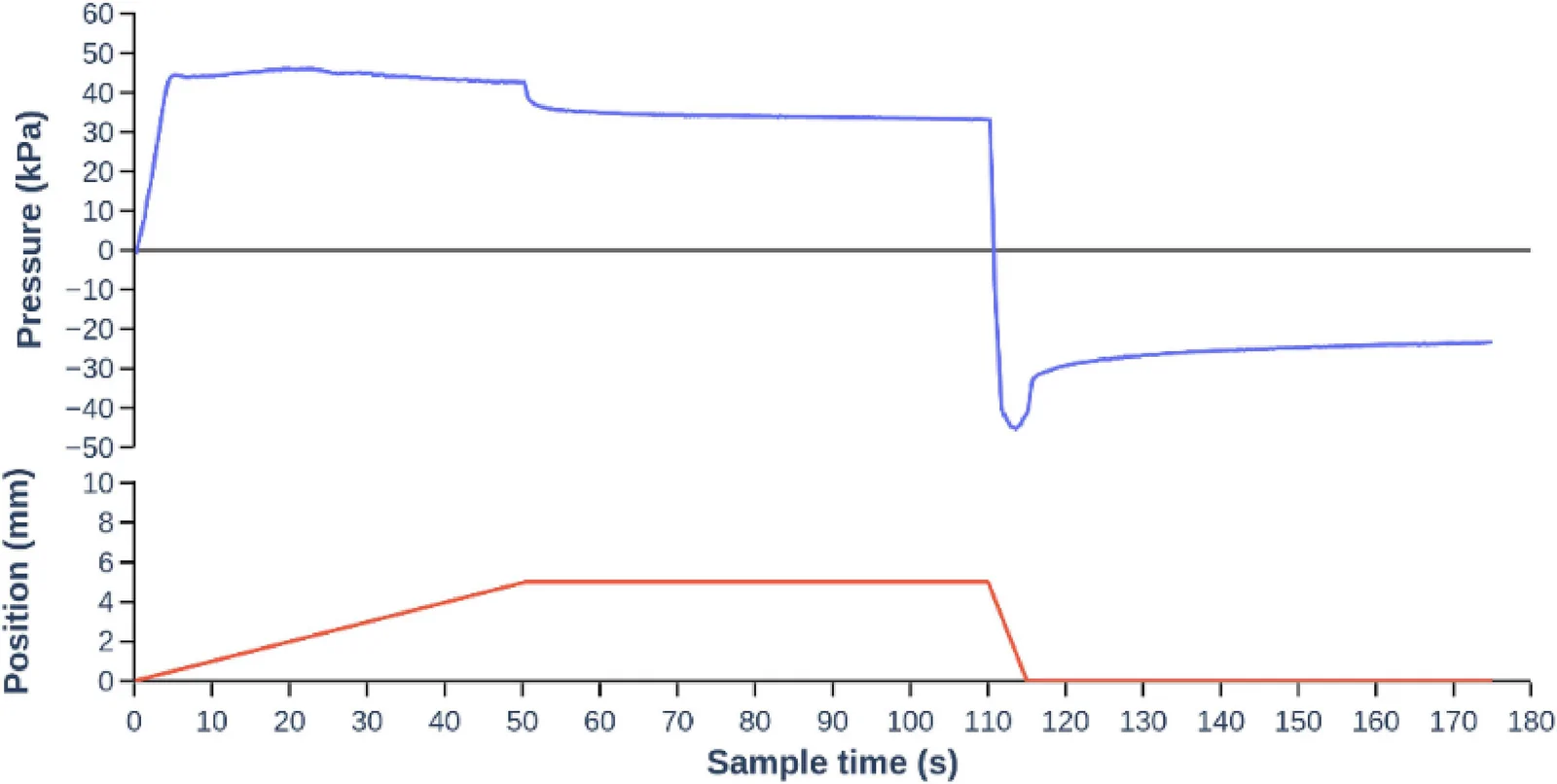
Graph of the extrudability profile of pharma-ink. The graphs show the relation between the displacement of the syringe plunger (red line) and the applied pressure (blue line).

The rheological properties of the pharma-ink were studied using the pressure sensor integrated in the SSE Laguna printhead (Fig. 2B**)**, facilitating the optimization of the printing parameters. Optimal printing conditions were achieved at a temperature of 60 °C and an extrusion rate of 50 mm/min. These parameters were selected because they provided the most stable rheological profile (Figure S1), resulting in constant pressure throughout the entire blister-filling process (Fig. 3). The pharma-ink required a threshold pressure of 45 kPa to initiate flow through the nozzle. Once this pressure was reached, a steady flow was maintained. During the holding period, the pressure remained constant at 35 kPa. Upon releasing the applied pressure, a sharp drop in pressure was observed, and the pharma-ink flow through the nozzle ceased rapidly (Fig. 3). Lower temperatures resulted in excessive viscosity, requiring higher threshold pressures, while higher extrusion rates compromised extrusion accuracy.

The relation between the plunger displacement and the weight registered by the balance determined an extrusion rate of 37.05 mg/mm. This value was then entered into the printing parameters of the pharma-ink to establish the dose library. The accuracy of this calibration was verified by gravimetric analysis of the printed troches, ensuring that the theoretical mass calculated from the plunger displacement aligned with the actual measured weight for each dose in the library.

Following the creation of the dose library, the 3D printer successfully printed troches across a dose range of 5 to 20 mg. The blister holder surface allowed simultaneous printing into four blisters per batch, enabling the production of up to 48 troches per run. In addition, the M3DIMAKER Studio™ software enabled printing different doses in each blister cavity or pocket within the same printing process, providing substantial flexibility and streamlining workflow. The blister-filling process was rapid (2 s per troche) and precise. An integrated quality control system was employed to ensure the quality of the dosage forms throughout the manufacturing process. No material loss between cavities was observed, and the filling of the four blisters (48 troches) was completed in less than 5 min, including the stabilization time of the integrated balance between each troche.

**Fig. 4:**
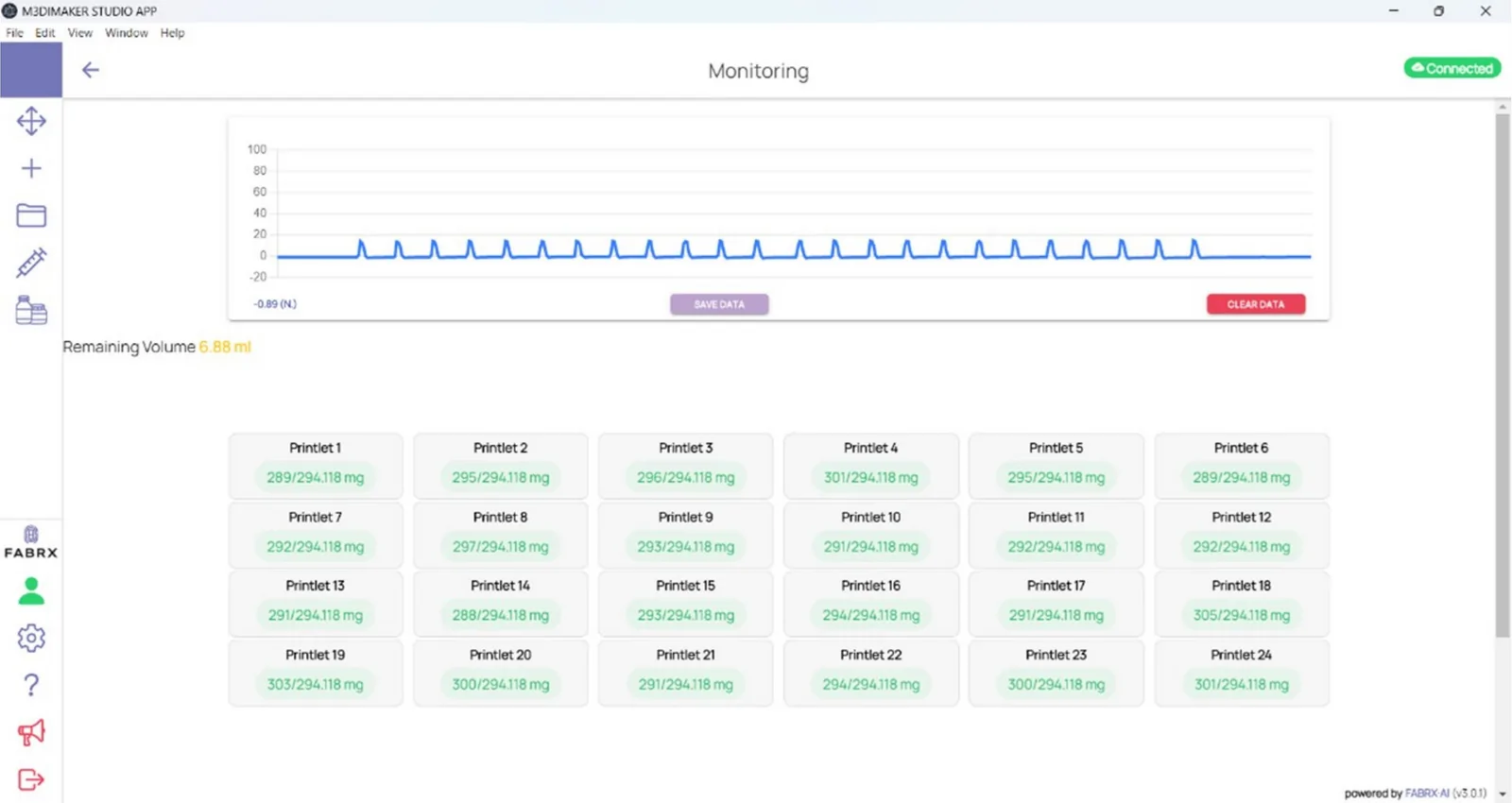
Screenshot of the M3DIMAKER Studio*™* software while printing 5 mg baclofen troches. The upper section shows a pressure chart that monitors the pressure required to extrude each troche. The lower section displays the weights of the 3D printed troches, indicating the target weight (right) and the actual measured weight (left).

The mass uniformity test was conducted in accordance with the requirements specified by the EP. According to these guidelines, no more than two units out of twenty may deviate from the mean mass by more than ± 5%. The printed troches exhibited average masses of 294.71 ± 4.71 mg, 591.52 ± 9.64 mg, and 1188.33 ± 17.06 mg for the 5 mg, 10 mg, and 20 mg doses, respectively. The M3DIMAKER Studio™ software, which controls the printer, enables real-time monitoring of troche weights during the printing process, allowing comparison between the actual and target weights. Furthermore, the integrated sensor measures the pressure required to extrude each dosage form, enabling the detection of issues such as air bubbles in the syringe or nozzle obstruction (Fig. 4). All printed troches met the acceptance criteria established by the EP.

On the other hand, the manual method resulted in significant material wastage between the mold cavities. This approach lacked the flexibility to produce different doses within the same mold, requiring either molds of different sizes or the development of dose-dependent formulations. Additionally, individual troches required manual weighing to verify their quality, increasing the labor intensity of the process.

For the manual troches, the average masses were 287.23 ± 9.20 mg, 516.63 ± 12.83 mg, and 1212.29 ± 27.39 mg for the 5 mg, 10 mg, and 20 mg doses, respectively. There were notable differences in the weights of the 10 mg troches. A key distinction between the manual process and the automated 3D printing method is that, in the former case, the cavities of the mold are fully filled with the formulation, and the final dose is determined by the cavity volume. In contrast, when using a 3D printer, the quantity of material deposited is automatically adjusted to achieve the target dose, independent of the container volume. Additionally, four manual troches from the 5 mg batch exceeded the permitted deviation, and therefore did not comply with the EP. Differences in mold geometry, filling process, packaging, sealing method, and operator handling may influence the properties of both manually and printed troches.

**Fig. 5:**
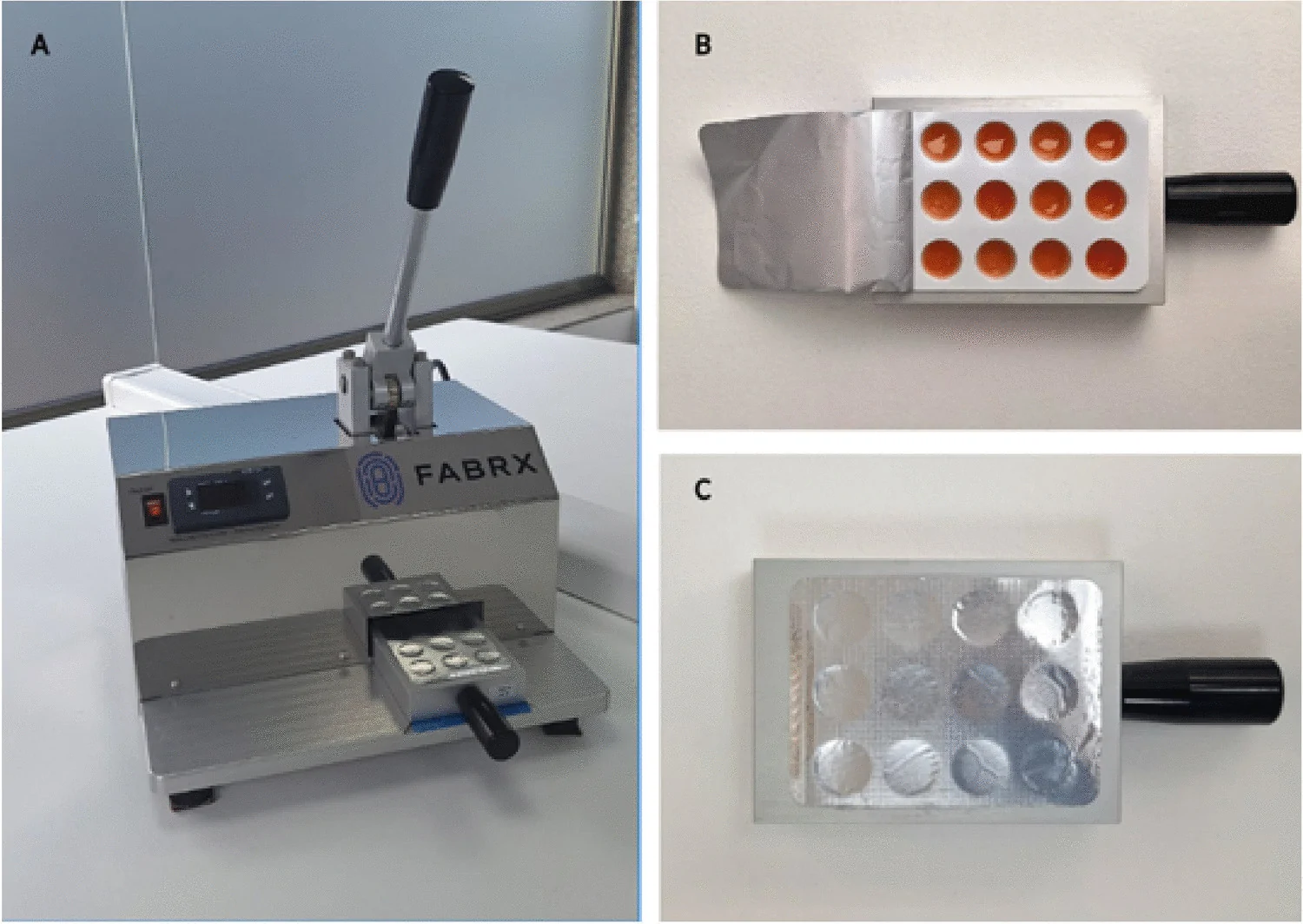
(**A**) Blister heat-sealing machine; (**B**) Unsealed blister containing liquid troches; (**C**) Sealed blister.

Following the printing process, the blisters were sealed. One of the advantages of the automatic blister-filling process compared with the traditional method is that packaging can be performed immediately after preparing the formulation. In this case, it was not necessary to wait for the troches to solidify prior to packaging, as each troche solidifies directly within its respective blister cavity (Fig. 5B). Furthermore, the cavities are never completely filled, thereby eliminating the risk of aluminum fusing with the troches during the sealing process. The result is a properly sealed blister, ready for storage or direct dispensing.

### Dimensions of the Troches

**Fig. 6:**
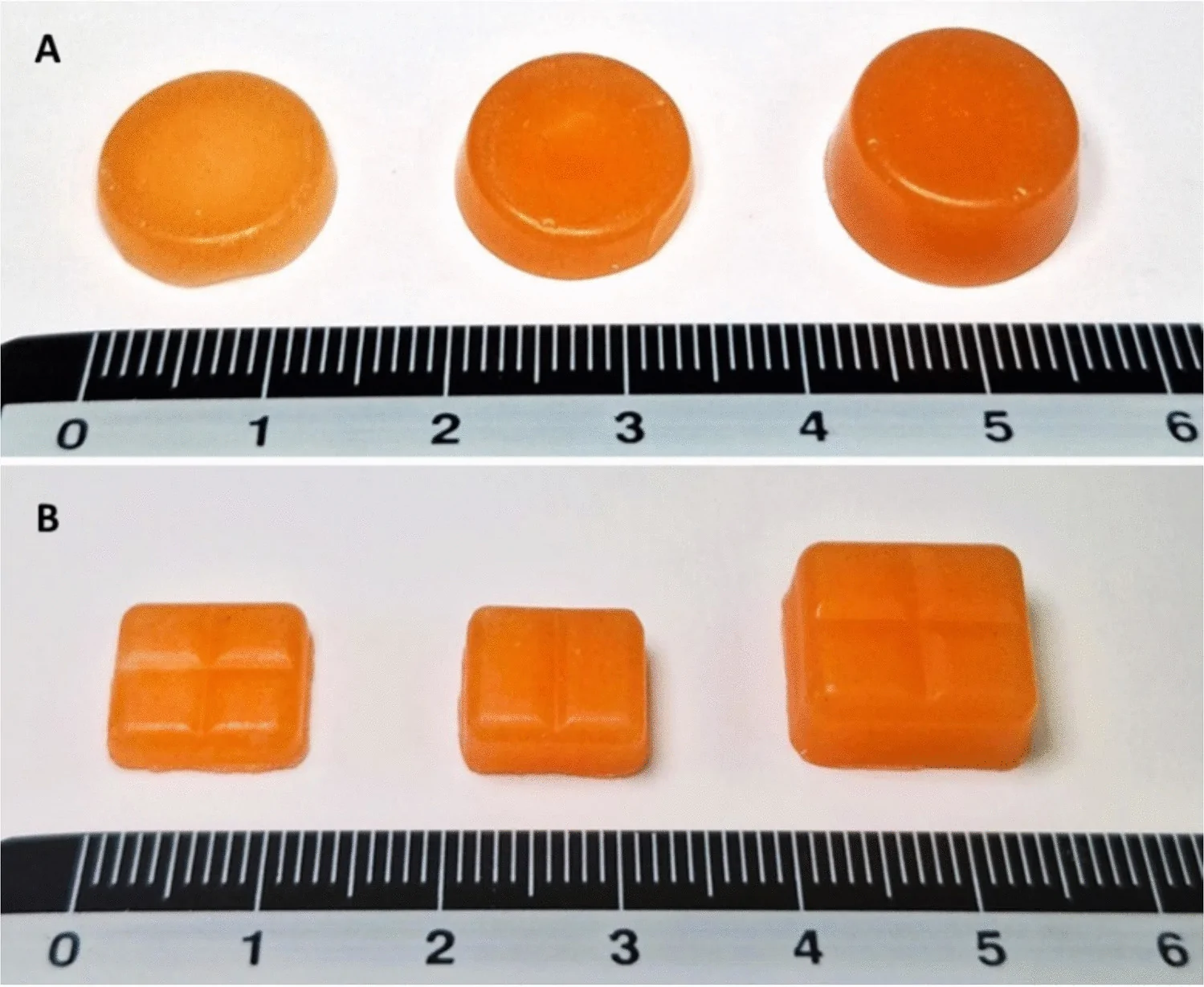
(**A**) Printed troches; (**B**) Manual troches prepared using molds of different volumes (from left to right 0.25 mL, 0.5 mL and 1 mL). The corresponding baclofen doses were 5, 10 and 20 mg, from left to right. Scale in cm.

The dimensions of the troches were determined according to their dose and preparation method. Due to the use of two different production surfaces (blister and molds), the troches exhibited distinct shapes (Fig. 6). Round blisters were used to facilitate dosage form packaging, improving traceability and workflow. Additionally, this shape has demonstrated good patient acceptability, which can increase treatment adherence [41]. In both types of troches, higher doses required larger troche sizes. The printed troches were produced using blisters with fixed diameters (14.5 mm upper diameter and 12.0 mm lower diameter), and only the height varied according to the dose. Heights of 3.0 mm, 5.0 mm, and 7.0 mm were obtained for the lowest, intermediate, and highest doses, respectively.

In the case of manually prepared troches, rectangular molds were used, as this was the compounding method routinely employed at the hospital. For the 5 mg dose, manual troches measured 3.0 mm in height, 9.0 mm in length, and 10.0 mm in width. For the 10 mg dose, length and width remained unchanged, while height increased to 5.0 mm. For the 20 mg dose, troche dimensions increased to 7.0 mm in height, 12.0 mm in length, and 14.0 mm in width.

### Friability Test

The results for the friability test are shown in Table I. As there are no specific requirements for this type of dosage form in the EP, the criteria established for uncoated tablets were applied (weight loss must be below 1%) [33]. Both formulations complied with the established criteria of the EP. An inverse relationship was observed between weight loss and dose, with lower doses exhibiting higher friability values. This behavior can be attributed to the reduction in troche size. Smaller troches have a greater surface area-to-volume ratio, which increases their susceptibility to erosion. Consequently, smaller troches exhibited higher friability compared to their larger counterparts [42].

**Table 1:** Results for friability test and drug content test.

Formulation	Friability (%)	Baclofen recovery (%) in drug content
5 mg printed troche	0.76	99.92 ± 1.67
10 mg printed troche	0.27	97.72 ± 1.80
20 mg printed troche	0.14	101.11 ± 0.74
5 mg manual troche	0.12	100.53 ± 0.63
10 mg manual troche	0.05	83.90 ± 2.46
20 mg manual troche	0.00	100.77 ± 2.12

The printed troches showed slightly higher weight loss than those prepared by the traditional method; however, all formulations complied with the European Pharmacopoeia (Ph. Eur.) requirements. The observed difference may be related to variations in geometry between the two types of formulations. As reported in the literature, geometries with sharper edges can act as points of stress concentration. When mechanical force is applied, stress may localize at these areas, increasing their susceptibility to surface erosion and, consequently, friability [43].

The conical geometry of the printed troches may differ from that obtained using molds for manual filling, which could account for the modest difference in weight loss. Nonetheless, the performance of all formulations remained within acceptable pharmacopoeial limits.

### X-ray Powder Diffraction Analysis (XRPD)

**Fig. 7:**
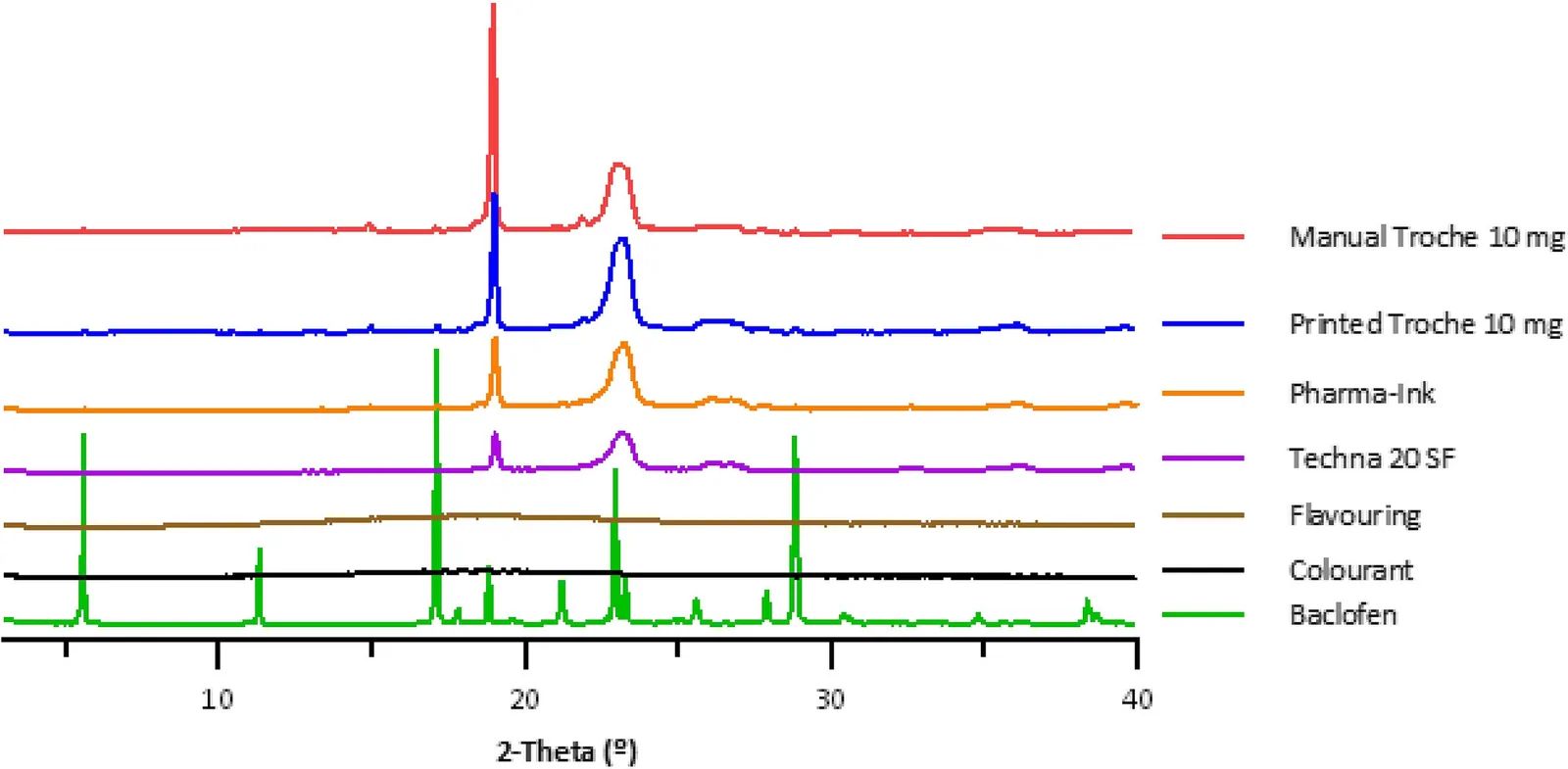
X-ray diffraction patterns of pure baclofen, excipients, pharma-ink, printed and manual troche.

The pattern for pure baclofen showed characteristic peaks at 2-Theta (θ) values of 5.60°, 17.11°, 18.81°, 21.20°, 23.27°, and 28.81° (Fig. 7). This demonstrates that the drug is at least partially in a crystalline state, in agreement with other studies [44]. On the other hand, Techna 20 SF™ exhibited broader peaks than baclofen, suggesting that it is in a semi-crystalline state [29]. In contrast, both the colorant and the flavoring agent did not show any well-defined peaks, indicating that they are in an amorphous state. Regarding the diffractograms corresponding to the pharma-ink and the troche, the presence of peaks associated with baclofen confirms that part of the drug is in a crystalline state.

### Thermal Analysis

**Fig. 8:**
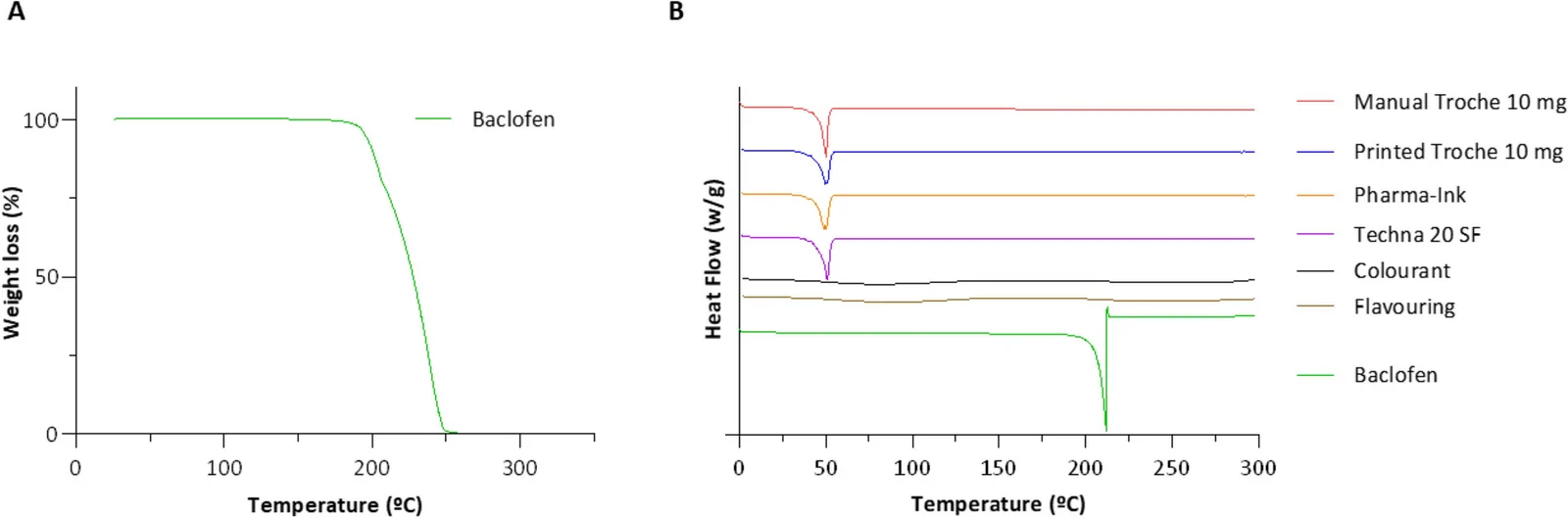
(**A**) TGA profile of pure baclofen; (**B**) DSC curves of baclofen, excipients, pharma-ink and troches.

The TGA was conducted to assess the thermal stability of baclofen (Fig. 8** A).** Weight loss was observed at temperatures above 180 °C, becoming more pronounced beyond 200 °C, indicating drug thermal degradation. These results confirm that baclofen remains stable at the temperature used during the troche manufacturing process.

Additionally, DSC was employed to characterize the solid-state form of the drug in the pharma-ink and troches (Fig. 8** B**). Baclofen showed a melting point of approximately 211.90 °C, while Techna 20 SF™ displayed a melting point at 50.82 °C, consistent with the literature [29, 45, 46]. The absence of endothermal peaks for the colorant and flavoring agent suggests their amorphous state. In the pharma-inks and troches, endothermal peaks corresponding to Techna 20 SF™ were detected; however, no signals attributable to baclofen were observed. This observation is likely attributable to the drug's solubility in PEGs, which are the main components of the Techna 20 SF™ base. Consequently, baclofen is presented as a solid dispersion within the polymeric matrix, a mechanism that has been previously documented in the literature for enhancing the solubility of poorly water-soluble drugs [47, 48].

### Determination of Drug Content

The results of the drug content of the troches were determined in accordance with the content uniformity requirements outlined in the EP. According to these guidelines, the drug content must fall within the range of 85% to 115%, with no individual unit exceeding the limits of 75% to 125% [49]. It was observed that the 10 mg manually prepared troches did not comply with the established requirements, achieving only 83.90 ± 2.46% recovery. This non-compliance was attributed to variability in the weight, which was consistently lower than the target weight. All other troche formulations showed a drug content within the range of 97.72–101.11%, thereby meeting the EP criteria (Table I).

### *In vitro* Disintegration Test

As there are no specific requirements for troches, the *in vitro* disintegration test was evaluated according to the requirements for uncoated tablets. Therefore, all troches must be completely disintegrated within 15 min [50].

**Fig. 9:**
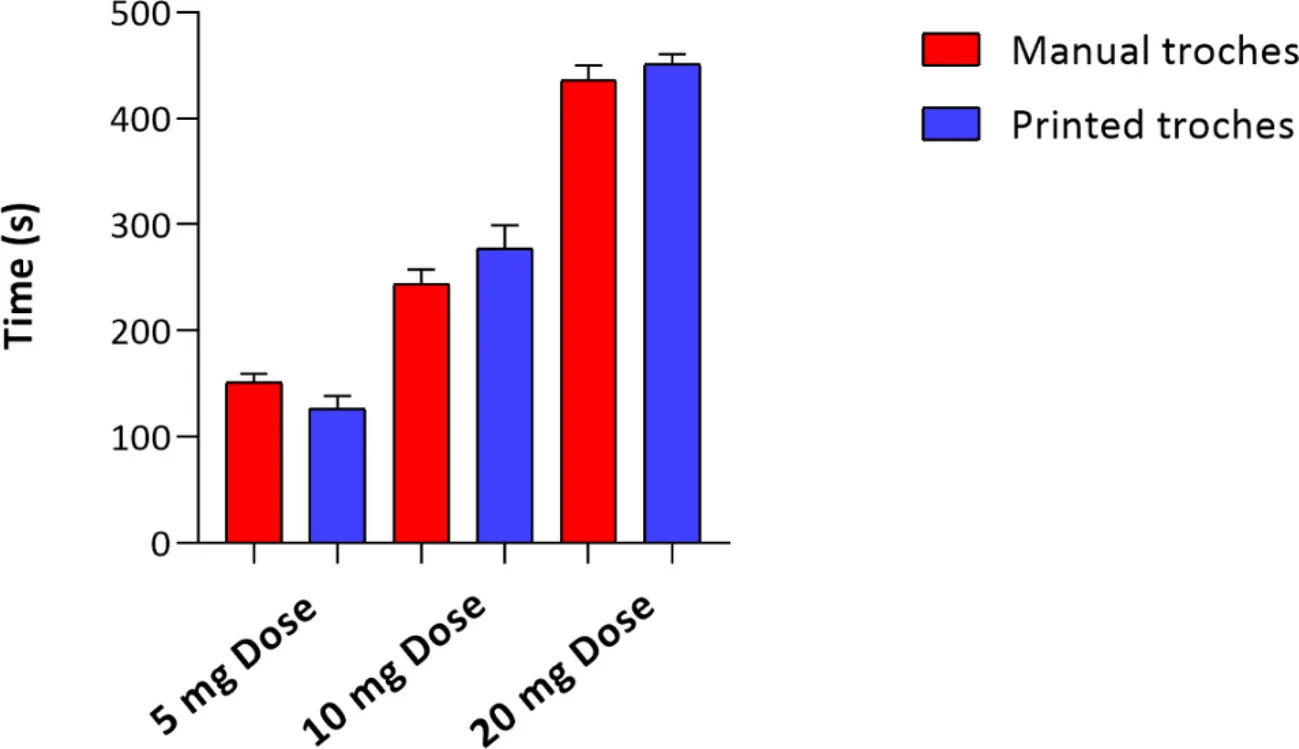
*In vitro* disintegration times for manual and printed baclofen troches.

The results showed that all troches, both manually prepared and printed, completely disintegrated in less than 8 min, with longer disintegration times observed as the dose increased. Consequently, all troches complied with the EP requirements. No significant differences were observed between the two types of formulations **(**Fig. 9).

### *In vitro* Dissolution Test

**Fig. 10:**
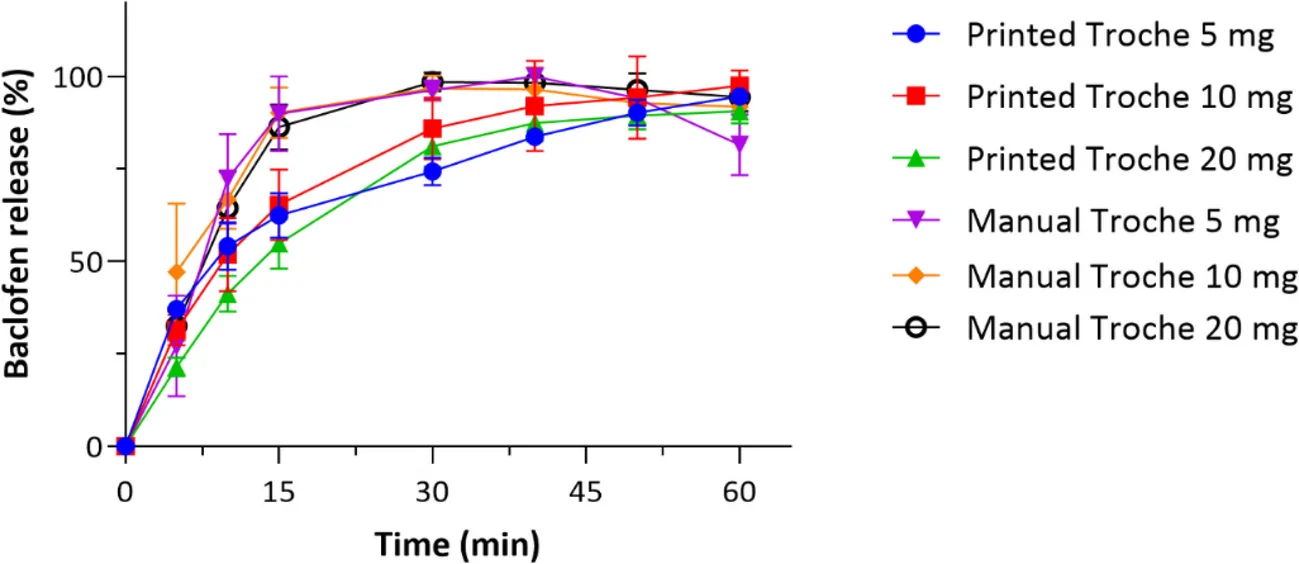
*In vitro* baclofen release profiles of printed troches in acidic medium (0.01 N HCl) at 37 °C and 50 rpm.

The dissolution test indicated that both manual and printed troches exhibited immediate drug release (Fig. 10). All troches released more than 75% of baclofen within 45 min, meeting the requirements of the EP [51]. Some differences were observed between the manual and the printed troches. Manually prepared troches exhibited a faster release rate of baclofen, reaching 100% release in 30 min. However, printed troches released 75 to 85% of the drug during the same period. This difference can be mainly attributed to the surface area-to-volume ratio, a critical factor on dissolution rate. This is consistent with previous studies, where prismatic geometries, such as prism or cubes, demonstrated higher dissolution rates than cylindrical shapes [52].

### Stability Test

A six-month stability study was conducted for both types of troches (Table II**)**. The printed troches remained stable throughout the study period, with no degradation peaks detected in the HPLC chromatograms. In contrast, the 10 mg manually prepared troches showed lower recovery values at the final sampling point, despite having the same composition. If the formulation itself were unstable, a consistent decrease in recovery would be expected across all samples. However, the recovery values of the remaining manually prepared troches were satisfactory, indicating that the formulation was stable. The observed variability is therefore likely attributable to differences in the packaging method. The manually prepared troches were sealed manually, making them more susceptible to inadequate sealing and reduced protection. In contrast, the printed troches were sealed using a blister heat-sealing machine, resulting in improved seal integrity and better preservation of the product [53].

**Table 2:** Stability study data for manual and printed troches.

Days	Average drug content (%)								
	Manual troches			Printed troches					
	5 mg dose	10 mg dose	20 mg dose		5 mg dose	10 mg dose	20 mg dose		
0	99.64 ± 0.40	98.61 ± 0.79	97.28 ± 0.35		99.59 ± 0.20	99.62 ± 1.35	101.35 ± 0.39		
15	101.86 ± 1.16	99.67 ± 0.85	100.49 ± 0.47		100.27 ± 2.14	103.64 ± 0.37	103.16 ± 1.36		
30	99.31 ± 1.69	98.04 ± 2.23	96.76 ± 1.23		100.10 ± 3.48	101.19 ± 0.69	98.51 ± 0.72		
60	97.38 ± 0.75	96.64 ± 1.09	98.32 ± 2.31		103.31 ± 1.94	100.33 ± 1.68	100.88 ± 2.22		
90	100.15 ± 1.38	101.89 ± 0.22	101.14 ± 1.74		98.44 ± 2.08	101.89 ± 0.22	101.14 ± 1.74		
120	99.63 ± 0.74	99.79 ± 0.75	97.75 ± 0.89		99.97 ± 1.09	99.49 ± 1.32	100.25 ± 0.57		
180	100.52 ± 1.41	91.67 ± 5.91	100.02 ± 0.75		99.95 ± 1.05	97.64 ± 2.01	100.74 ± 2.53		

### Preparation and Dispensing of Baclofen Troches Using a Pharmaceutical 3D Printer as a Blister Filling Platform in a Pharmacy Hospital

The M3DIMAKER 1™ printer was successfully installed in the Pharmacy Department of the General University Hospital Doctor Balmis in Alicante, a hospital recognized as a leading healthcare institution in Spain. FABRX technical team conducted the IQ/OQ/PQ and the SAT of both the printer and the M3DIMAKER Studio™ software, to ensure proper functioning and compliance with Good Manufacturing Practice (GMP) requirements.

Different batches of baclofen troches at doses of 5, 10, and 20 mg were prepared for three months, in accordance with the corresponding SOPs. These are precise protocols that specify all the steps required to ensure the correct manufacturing of the troches. Quality control was performed on all the troches produced, using the quality systems integrated into the printer. All data generated during the printing process were recorded by the printer’s software, facilitating full traceability of the manufactured batches. The produced formulations were dispensed to four patients in accordance with their prescriptions, with all patients successfully completing the treatment with no reported issues.

A labeling system incorporating QR codes was implemented in the M3DIMAKER Studio™ to enhance traceability and ensure the secure identification of the formulations. Each syringe was labeled with a QR code that linked directly to the printer’s software, providing immediate access to critical information such as composition, batch number, and specific printing parameters. This robust identification system supports full traceability and quality assurance across the entire manufacturing process, minimizing the risk of human error.

## Conclusions

For the first time, different doses of baclofen troches were successfully prepared in a hospital pharmacy service using a pharmaceutical 3D printer equipped with a dual advanced quality control system. This study not only demonstrates the quality and consistency of medicines produced using this technology, but also confirms the feasibility of employing 3D printing for the automated manufacturing of troches through automatic blister filling, improving traceability and facilitating workflow at dispensing points.

Techna 20 SF™ was selected for its well-established safety profile in pharmaceutical compounding and it enabled rapid pharma-ink production while streamlining the compounding process for pharmacists. The results obtained from mass uniformity and drug content analyses confirmed that the printed troches contained an accurate dose, in contrast to those produced using the manual method. In addition, *in vitro* dissolution and disintegration tests demonstrated immediate drug release in the oral cavity. Finally, stability testing confirmed the viability of the printed troches for up to six months.

This study demonstrates the feasibility of integrating a pharmaceutical 3D printer into a hospital pharmacy setting, enabling automated production with built-in quality control systems. Additionally, it establishes the technological and operational advantages of the 3D printing approach compared with traditional manual methods. By allowing the production of medicines with significantly higher dose accuracy and reproducibility, the automated system reduces manufacturing risks, thereby contributing to enhanced patient safety through rigorous process control. Furthermore, the formulations were administered to patients, reflecting a complete, real-world workflow from clinical need to final administration. Altogether, this represents a significant step forward in the practical implementation of 3D printing technologies in clinical environments, supporting the development of potentially safer, faster, and more personalized medication manufacturing processes.

## Supplementary Information

Below is the link to the electronic supplementary material.

ESM 1 DOCX (177 KB)
