# Supplementary material for: On-Demand Hospital Manufacturing of Baclofen Lozenges Using Pharmaceutical 3D Printing
Source: Pharm Res. 2026 Aug 11;43(9):3411–25. doi: 10.1007/s11095-026-04164-7 (PMC13642443; doi:10.1007/s11095-026-04164-7)
Supplement: Supplementary file 1 [file 11095_2026_4164_MOESM1_ESM.docx]

**Title:** *On-Demand Hospital Manufacturing of Baclofen Lozenges Using Pharmaceutical 3D Printing.*

**Authors**: *Daniel Grandal-Gonzalez^1^, Carlos Bendicho-Lavilla^2,3^, Eduardo Diaz-Torres^2,3^, Rocio Sanin-Fontela^1^, María L. Couce^4^, María José de Castro-López^4^, Isabel Gonzalez-Alvarez^5^, Marta Gonzalez-Alvarez^5^, Alejandro Ruiz-Picazo^5^, Marival Bermejo^5^, Pedro Garcia-Salom^6^, Abdul W. Basit^2,3,7^, Carmen Alvarez-Lorenzo^1, *^, Alvaro Goyanes^1,2,3,7,^* ^*^

**Affiliations:***^1^ Departamento de Farmacología, Farmacia y Tecnología Farmacéutica, I+D Farma (GI-1645), Facultad de Farmacia, Instituto de Materiales (iMATUS) and Health Research Institute of Santiago de Compostela (IDIS), Universidade de Santiago de Compostela, 15782 Santiago de Compostela, Spain.*

*^2^ FABRX Artificial Intelligence, Calle Enrique Vidal Abascal 7, 15702, Santiago de Compostela, Spain.*

*^3^ FABRX Ltd., Henwood House, Henwood, Ashford, Kent, TN24 8DH, UK.*

*^4^ Servicio de Neonatología, Unidad de Diagnóstico y Tratamiento de Enfermedades Metabólicas Congénitas, Health Research Institute of Santiago de Compostela (IDIS), Hospital Clínico Universitario de Santiago de Compostela, Universidad de Santiago de Compostela, IDIS, RICORS, CIBERER, MetabERN, Spain*

*^5^ Department of Pharmacokinetics and Pharmaceutical Technology, Miguel Hernandez University, San Juan de Alicante, 03550 Alicante, Spain*

*^6^ Pharmacy Department, University Clinical Hospital Dr. Balmis, 03010 Alicante, Spain*

*^7^ Department of Pharmaceutics, UCL School of Pharmacy, University College London, 29-39 Brunswick Square, London, WC1N 1AX, UK.*

**Correspondence**: *C.A.L. carmen.alvarez.lorenzo@usc.es; A.G. a.goyanes@fabrx.co.uk*

**Journal:** *Pharmaceutical Research*

**S1. Rheological characterization of the pharma-ink**

Rheological characterization of the pharma-ink was performed at 60°C, in duplicate, using a Rheolyst AR1000-N rheometer (TA Instruments, Delaware, USA) equipped with an AR2500 data analyzer and a Peltier plate temperature control. A 40 mm diameter parallel steel plate was used as the measuring element (gap 1000 µm). Aliquots of each pharma-ink were subjected to flow tests, applying controlled shear rate rotational viscometry covering a wide range of values, as detailed below. The pharma-inks were previously heated to the same temperature in the preheater of the printer to simulate the conditions of the printing process. Samples of the formulation were directly extruded onto the platform of the rheometer. The experimental protocol consisted of a continuous shear rate sweep operating in linear mode, divided into two consecutive steps: an upward ramp (Step 1) from 0.05 to 10 s⁻¹ and a downward ramp (Step 2) from 10 to 0.05 s⁻¹. Each step was performed over a duration of 5 minutes, collecting 200 sampling points per ramp to quantify the viscosity of the formulation as a function of shear rate.

The pharma-inks showed a yield stress of 0.06 Pa, beyond which they flowed showing an almost constant viscosity, with values of 0.11-0.13 Pa·s, in the shear rate range evaluated (**Figure S1)**. Additionally, both shear ramps display an almost complete superposition. The absence of a hysteresis loop demonstrates that the formulation does not possess thixotropic properties.

This specific rheological profile is highly advantageous for blister-filling technology. Operating at 60°C maintains the formulation in a fully liquid state with a constant, low viscosity. Furthermore, the absence of thixotropic behavior ensures that the formulation responds instantaneously to the pressure-driven extrusion and retraction movements, effectively eliminating any material dripping and inherently weight and dose errors.

**Figure S1**. Viscosity of the pharma-ink as a function of the shear rate.
